# Conductive Collagen-Based Hydrogel Combined With Electrical Stimulation to Promote Neural Stem Cell Proliferation and Differentiation

**DOI:** 10.3389/fbioe.2022.912497

**Published:** 2022-06-17

**Authors:** Xinzhong Xu, Lin Wang, Juehua Jing, Junfeng Zhan, Chungui Xu, Wukun Xie, Shuming Ye, Yao Zhao, Chi Zhang, Fei Huang

**Affiliations:** ^1^ Department of Orthopaedics, The Second Affiliated Hospital of Anhui Medical University, Hefei, China; ^2^ Department of Orthopaedics, Yijishan Hospital of Wannan Medical College, Wuhu, China; ^3^ Department of Orthopaedics, The Fourth Affiliated Hospital of Anhui Medical University, Hefei, China

**Keywords:** electrical stimulation, collagen, polypyrrole, conductive hydrogel, neural stem cells

## Abstract

Injectable biomimetic hydrogels are a promising strategy for enhancing tissue repair after spinal cord injury (SCI) by restoring electrical signals and increasing stem cell differentiation. However, fabricating hydrogels that simultaneously exhibit high electrical conductivities, excellent mechanical properties, and biocompatibility remains a great challenge. In the present study, a collagen-based self-assembling cross-linking polymer network (SCPN) hydrogel containing poly-pyrrole (PPy), which imparted electroconductive properties, is developed for potential application in SCI repair. The prepared collagen/polypyrrole (Col/PPy)-based hydrogel exhibited a continuous and porous structure with pore sizes ranging from 50 to 200 μm. Mechanical test results indicated that the Young’s moduli of the prepared hydrogels were remarkably enhanced with PPy content in the range 0–40 mM. The conductivity of Col/PPy40 hydrogel was 0.176 ± 0.07 S/cm, which was beneficial for mediating electrical signals between tissues and accelerating the rate of nerve repair. The investigations of swelling and degradation of the hydrogels indicated that PPy chains interpenetrated and entangled with the collagen, thereby tightening the network structure of the hydrogel and improving its stability. The cell count kit-8 (CCK-8) assay and live/dead staining assay demonstrated that Col/PPy40 coupled with electrical simulation promoted the proliferation and survival of neural stem cells (NSCs). Compared with the other groups, the immunocytochemical analysis, qPCR, and Western blot studies suggested that Col/PPy40 coupled with ES maximally induced the differentiation of NSCs into neurons and inhibited the differentiation of NSCs into astrocytes. The results also indicated that the neurons in ES-treated Col/PPy40 hydrogel have longer neurites (170.8 ± 37.2 μm) and greater numbers of branch points (4.7 ± 1.2). Therefore, the prepared hydrogel system coupled with ES has potential prospects in the field of SCI treatment.

## Introduction

Stem cell transplantation therapy has been considered a promising method to replace missing cells in a damaged spinal cord to stimulate neuroprotection and nerve regeneration (Seif et al., 2020). Through the transformation and transplantation of neural stem cells (NSCs), the recovery of spinal cord nerve tissue was effectively promoted (Sahni and Kessler, 2010). Transplanted NSCs in the injury site released a series of neurotrophic growth factors and differentiated into neurons to improve neurogenesis and axonal growth (Lu et al., 2003). Although great progress has been made, the poor microenvironment remains the biggest obstacles to the clinical applications of NSC-based therapies (Cofano et al., 2019; David et al., 2019).

Electrical activity has been shown to play an important role in neural development by regulating signal transmission (Marquez-Chin and Popovic, 2020). In terms of preclinical studies with NSCs, electrical stimulation (ES) has also been proven to alter NSCs transcriptome, including changes to the VEGF-A pathway and genes involved in cell proliferation, inflammatory response, and synaptic remodeling (George et al., 2017). Several studies revealed that ES can increase neurite outgrowth *in vitro* and enhance functional recovery *in vivo* (Gordon, 2016; Imaninezhad et al., 2018; Koppes et al., 2016). Therefore, it is urgent to develop bioelectronic tissue-engineering materials to manipulate the stem cell microenvironment (Kiyotake et al., 2022; Leppik et al., 2020).

Recently, conductive biomaterials are of particular interest in neuroscience because they allow for the application of electrical stimulation (ES) (Ghasemi-Mobarakeh et al., 2009; Zhu et al., 2018; Yan et al., 2020). Conductive hydrogels are conductive materials capable of absorbing and retaining large amounts of biological fluids (Guo and Ma., 2018); they have been widely used in smart electronic devices, biosensors, nerve repair engineering, and other fields (Balint et al., 2014; Sirivisoot et al., 2014; Lu et al., 2018; Zhao and Ma., 2018; Ghasemi-Mobarakeh et al., 2011). In nerve repair applications, the advantage of conductive hydrogel is that it can provide both physical and electrical properties, in which the former is the unique property of the hydrogel and the latter is the conductivity performed by the conductive materials. Collagen is an important component of the extracellular matrix (ECM) and has been widely studied in three-dimensional (3D) cell culture because of its good biocompatibility, biodegradability, and cell adhesion (Ge et al., 2013). For example, in our previous study, collage gel has been proven to provide an appropriate environment to support rat NSCs adhesion and proliferation (Huang et al., 2013). By changing the degree of cross-linking, collagen can be made into different forms of microstructural scaffolds with controllable strength, porosity, and degradability. Considering these characteristics, it is more promising to biomimetically construct an electroconductive matrix based on collagen hydrogel for applications in neural regeneration (Sirivisoot et al., 2014).

Conductivity can be rendered to the hydrogels by incorporation of metal nanoparticles, carbon-based materials, and conductive polymers. Metal nanoparticles and carbon-based materials are widely used in the preparation of conductive biomaterials. For instance, Zhu et al. reported the integration of the conducting carbon nanofibrous scaffold and electrical stimulation can promote the NSCs proliferation. Rahimzadegan et al. demonstrated Au ions in polycaprolactone/chitosan scaffold can enhance the attachment and proliferation of fibroblast cells, and differentiation potential of mesenchymal stem cells toward neuron-like cells (Rahimzadegan et al., 2021). However, their long-term cytotoxicity and inhomogeneous distribution of the conducting particles in composite system restricted their widespread and effective use (Nezakati et al., 2018). Conductive polymers exhibited promising conductivity as bioactive scaffolds for neural regeneration and their conductive nature allows neural cells cultured on them to be stimulated by electrical signals. As the most studied conductive polymer, polypyrrole (PPy) has been widely used as a conductive component in conductive hydrogels due to its higher electrical conductivity and chemical stability than the other conductive polymers such as poly(3,4-ethylenedioxythiophene) (PEDOT) and polyaniline (PANI) (Wang et al., 2021; Zhao et al., 2021). PPy can also enhance cell adhesion, proliferation, and differentiation with or without ES (Gilmore et al., 2009).

We hypothesized that an electroconductive environment under 3D extracellular matrix (ECM) conditions could provide biochemical and biophysical cues for enhancing neurogenesis. In this study, we prepared a collagen-based self-assembling cross-linking polymer network (SCPN) hydrogel. The SCPN hydrogel utilized the self-assembly of collagen and chemical cross-linking reaction between collagen and PPy to form a network. This method effectively improved the mechanical properties of collagen-based hydrogel and prolonged their degradation time. Under stimulation by an electric current, the hydrogel enhanced the proliferation, survival, and differentiation of NSCs. The prepared SCPN hydrogel system has potential prospects in the field of neural tissue engineering.

## Materials and Methods

### Materials

Type I collagen (Mw > 3 × 10^5^ Da) was purchased from Guangzhou Tina Biotechnology Co., Ltd. PPy (Mw = 67 Da). Hydrochloric acid, anhydrous ferric chloride, sodium chloride, potassium chloride, potassium dihydrogen phosphate, anhydrous ethanol, and genipin were chemical grade and purchased from Sinopharm Group Chemical Reagent Co., Ltd.

### Preparation of Self-Assembling Cross-Linking Polymer Network Hydrogels

A homogeneous solution of collagen (10 mg/ml) was prepared in a 37°C DI water bath. Then, 1 ml of a pyrrole aqueous solution (concentrations of 0 mM, 20 mM, 40 mM, or 60 mM) was mixed with 1 ml of a collagen solution (10 mg/ml). Ferric chloride oxidant (molar ratio of pyrrole to ferric chloride 1:4) was slowly added to the mixed solution, thus initiating the polymerization of pyrrole monomers. After stirring for 30 min, PBS solution (pH = 7.4) was added dropwise to initiate collagen self-assembly. After 30 min of self-assembly, the mixed solution was soaked in a genipin solution (0.3 wt%). The cross-linking treatment was carried out at 37°C for 24 h. The prepared hydrogel was then washed with distilled water to remove residual genipin. The enhanced cross-linked collagen/PPy hydrogels were obtained, namely, Col/PPy0, Col/PPy20, Col/PPy40, and Col/PPy60.

### Characterizations of Self-Assembling Cross-Linking Polymer Network Hydrogels

The cross-sectional morphologies of the prepared hydrogels were examined using scanning electron microscopy (SEM; JSM-5600, JEOL, Tokyo, Japan) operated at 20 kV acceleration voltage. Before observation, the hydrogels were freeze-dried at −80°C for 24 h, fractured using a blade to obtain a dried hydrogel sheet, and then Au was coated with an approximately 30 nm thick coating layer. The mechanical strengths of Col/PPy hydrogel (diameter: 25 mm, thickness: 2 mm) were measured using a universal testing machine (model 5543; Instron, Norwood, MA, United States). Each sample was compressed with a 5 N load cell at a rate of 1 mm/min. The Young’s modulus of the hydrogel sample was obtained from the initial slope of the compressive stress-strain curve. The conductivity (unit: S/cm) of hydrogels (Col/PPy0, Col/PPy20, Col/PPy40, and Col/PPy60) was measured using a four-probe instrument.

### *In Vitro* Degradation and Swelling Properties of Self-Assembling Cross-Linking Polymer Network Hydrogels

The sample (10 mg) was put into a test tube, and the initial sample mass was recorded as W_0_. Collagenase solution (1 U/ml@2 ml) was added to the tube to immerse the sample, and the Eppendorf tube was kept at 37°C for degradation for 28 days. At different time points, the hydrogel was removed from the tube, and the solution was gently aspirated. The sample was then placed in a refrigerator (−20°C) for 5 h, followed by lyophilization for 1–2 days. After freeze-drying, the sample was weighed, and the degraded sample mass (W_1_) was recorded. The degradation ratio of the sample was calculated as follows:

$$\text{Degradation }\mathrm{ratio}\left(\text{\%}\right)=\left({\text{W}}_{\text{0}}-{\text{W}}_{1}\right)/{\text{W}}_{\text{0}}\times 100\text{\%}\text{.}$$

The sample (10 mg) was put into a test tube, and the initial sample mass was recorded as W_0_. Hydrogels were added into a centrifuge tube containing 20 ml PBS (pH = 7.4) at 37°C. At different time points, the hydrogels were taken out, and the weight was measured after the removal of excess water on the surface. The swelling ratio was calculated as follows:

$$\text{Swelling ratio }\left(\text{\%}\right)=\left({\text{W}}_{\text{t}}-{\text{W}}_{\text{0}}\right)/{\text{W}}_{\text{0}}\times 100\text{\%}\text{.}$$

### Cell Culture and Identification

NSCs were isolated and cultured as previously described (Huang et al., 2016). All rats used in the experiments were approved by the Institutional Animal Care and Use Committee of Anhui Medical University. Briefly, adult pregnant SD rats were anesthetized and killed with 100% CO_2_, and the neocortices of embryos (embryonic stage E15–16 days) were dissected, cut into small pieces, and mechanically triturated in cold PBS. The dissociated NSCs were seeded on low-attachment dishes and expanded in a maintenance medium containing DMEM/F12 (Gibco, United States), B27 neural supplement (2%, Gbico, United States), 2 mM GlutaMAX (Sigma–Aldrich, United States), 20 ng/ml epidermal growth factor (EGF, PeproTech, United States), and 20 ng/ml basic fibroblastic growth factor (bFGF, PeproTech, United States). The NSCs were cultured for 7 days. The obtained neurospheres were passaged three or four times and used for further experiment.

For identification of NSCs, neurospheres were placed on coverslips coated with 0.1% polylysine and nestin (a marker of NSCs) using an immunocytochemical method. For *in vitro* differentiation, a differentiation medium containing DMEM/F12 and 2% B27 was added, and the neurospheres were differentiated for 7 days. After differentiation, the neurons and astrocytes were identified by immunocytochemistry.

### Electrical Stimulation on the Neural Stem Cells Encapsulated in Hydrogels

After the NSCs were encapsulated in hydrogels for 24 h, the hydrogels were taken out and ES was performed on a voltage resource (Suing, Guangdong, China), as previously reported (Fu et al., 2009). First, a pair of L-shaped platinum electrodes separated by 10 mm were sterilized in 70% ethanol and UV light. Then, the electrodes were placed in 24-well culture plates to produce an electric field. The square-wave DC pulses stimulation of different current strengths was exerted onto the cells. After ES and another 12 h of culturing, the cells in different groups were studied further by CCK-8, live/dead staining, immunocytochemical analysis, qPCR, and Western bolt.

### Cell Proliferation Experiments Under Different ES Conditions

The CCK-8 assay (Dojindo Laboratories, Kumamoto, Japan) was used to evaluate the proliferation ability of NSCs in the prepared hydrogels under different voltage stimulation. The output voltage of the power supply was 0 mV, 100 mV, 200 mV, and 300 mV, respectively.

The ES was performed for 30 min every day. NSCs were inoculated on the surface of Col/PPy hydrogels at a cell density of 4 × 10^4^ cells/per hydrogel. The cells with the pure medium were set as the control group. The CCK-8 reagent was added on day 1, day 3, and day 7. After being cocultured for 3 h, the samples were drawn to measure the absorbance at 450 nm with a microplate reader (Multiskan FC, Thermo, United States).

### Cell Viability Assay

The viability of NSCs in different experimental groups was detected using a live/dead staining assay (Life Technologies, United States). A 24-well cell culture plate was used for detection, and a density of approximately 5 × 10^4^ cells/per hydrogel was seeded on different hydrogels and cultured in the differentiation medium for 3 days. For the ES treatment (Col/PPY40 + ES) as a positive group, the electrical stimulation (200 mV) was performed for 30 min every day, while Col/PPy0 and Col/PPy40 were set as negative groups. The differentiated cells were treated with 2 μM Calcein AM and 4 μM Ethidium homodimer I (EthD-1) for 30 min at room temperature and then washed three times with PBS. The images were taken at four random points per well using a confocal microscope (TCSSP8, Leica, Germany). All samples were studied in triplicate. The cell viability was calculated by dividing the live cell number over the total cell number.

### Immunocytochemical Assay

The NSCs were cultured on hydrogels with and without ES for 3 d and 7 days in a differentiation medium. The differentiation potential of incubation of NSCs was detected using an immunocytochemical assay. The cells were fixed with pre-chilled 4% paraformaldehyde for 20 min at room temperature, followed by PBS containing 5% normal goat serum and 0.3% Triton X-100. After 1 h, the primary antibodies of the neural stem cell marker nestin (1:250; Abcam, Shanghai, China), neuron marker β-tubulin III (1:100; Abcam, Shanghai, China), and glial cell marker GFAP (1:400; Abcam, Shanghai, China) were added and incubated at 4°C. After overnight incubation, the corresponding fluorescence secondary antibody was added and incubated at room temperature for 1 h. The nuclei were stained by adding DAPI, and the PBS was washed three times between each step. The observation was performed under a confocal microscope (TCSSP8, Leica, Germany). Twenty fields of view were randomly selected for each group, and the length of neuronal axons and number of protruding branch points of all neurons were recorded. The cells in the ES treatment group (Col/PPY40 + ES) were set as positive groups, the electrical stimulation (200 mV) was performed for 30 min every day, while those in Col/PPy0 and Col/Ppy40 groups were set as negative groups.

### Gene Expression Analysis

Quantitative real-time PCR (qRT-PCR) was used to detect the mRNA levels of related neural genes in the samples. After the cell groups were induced to differentiate for 3 d and 7 days, the culture medium was removed and rinsed three times with PBS. The prepared hydrogel was hydrolyzed with 1 mg/ml collagenase to release the 3D cultured cells. The released cells were lysed with TRIzol to collect the total intracellular RNA. The total RNA (1 mg) per group was reverse-transcribed into cDNA using the Reverse Transcription Kit. The gene expression levels of nerve cell-related β-tubulin III and GFAP were detected using Maxima SYBR Green qPCR Master Mix on a real-time qPCR detection system. The upstream and downstream primers used are listed in Table 1. The relative expression level of each target gene was calculated using the 2^−ΔΔCt^ method, and the expression levels of all target genes were relative to the reference gene GAPDH.

**TABLE 1:** Primers for qRT-PCR.

Genes	Forward primer	Reverse primer
β-tubulin III	TCA​CGC​AGC​AGA​TGT​TCG​AT	GTGGCGCGGGTCACA
GFAP	CCT​GAG​AGA​GAT​TCG​CAC​TCA​A	CTC​CTC​TGT​CTC​TTG​CAT​GTT​ACT​G

### Western Blot Analysis

β-tubulin III and GFAP protein expressions of differentiated NSCs on hydrogels with and without ES were also quantified using the Western blot analysis. The total protein concentration of cell lysates was measured by the BCA method using the BCA protein kit (Sangon Biotech, Shanghai, China). Forty micrograms of proteins were separated by 12% sodium dodecyl sulfate-polyacrylamide gel electrophoresis and transferred to polyvinylidene fluoride membranes (Sango Biotech, Shanghai, China). The membranes were blocked with 5% non-fat milk for 2 h and incubated with primary antibodies against β-tubulin III (1:100; Abcam, Shanghai, China), GFAP (1:200; Abcam, Shanghai, China), and GAPDH (1:1000; Abcam, Shanghai, China). After incubation for 3 h at room temperature, followed by 4°C overnight, the membranes were incubated with secondary antibody (Beyotime Institute of Biotechnology, Shanghai, China) at room temperature for 1 h and visualized using the ECL system (GE Healthcare Bio-Sciences AB, Image Quant LAS 500, Sweden).

### Statistics Analysis

Data were collected in triplicate and expressed as mean ± standard deviation using SPSS 22.0 software. One-way analysis of variance (ANOVA) combined with LSD post hoc tests were used to determine the level of significance. In all statistical evaluations, a probability (p) value of <0.05 (*) was considered significant, and a *p* value of <0.01 (**) was regarded as highly significant.

## Results and Discussion

### Characterizations of Various Collagen/Polypyrrole Hydrogels

**FIGURE 1:**
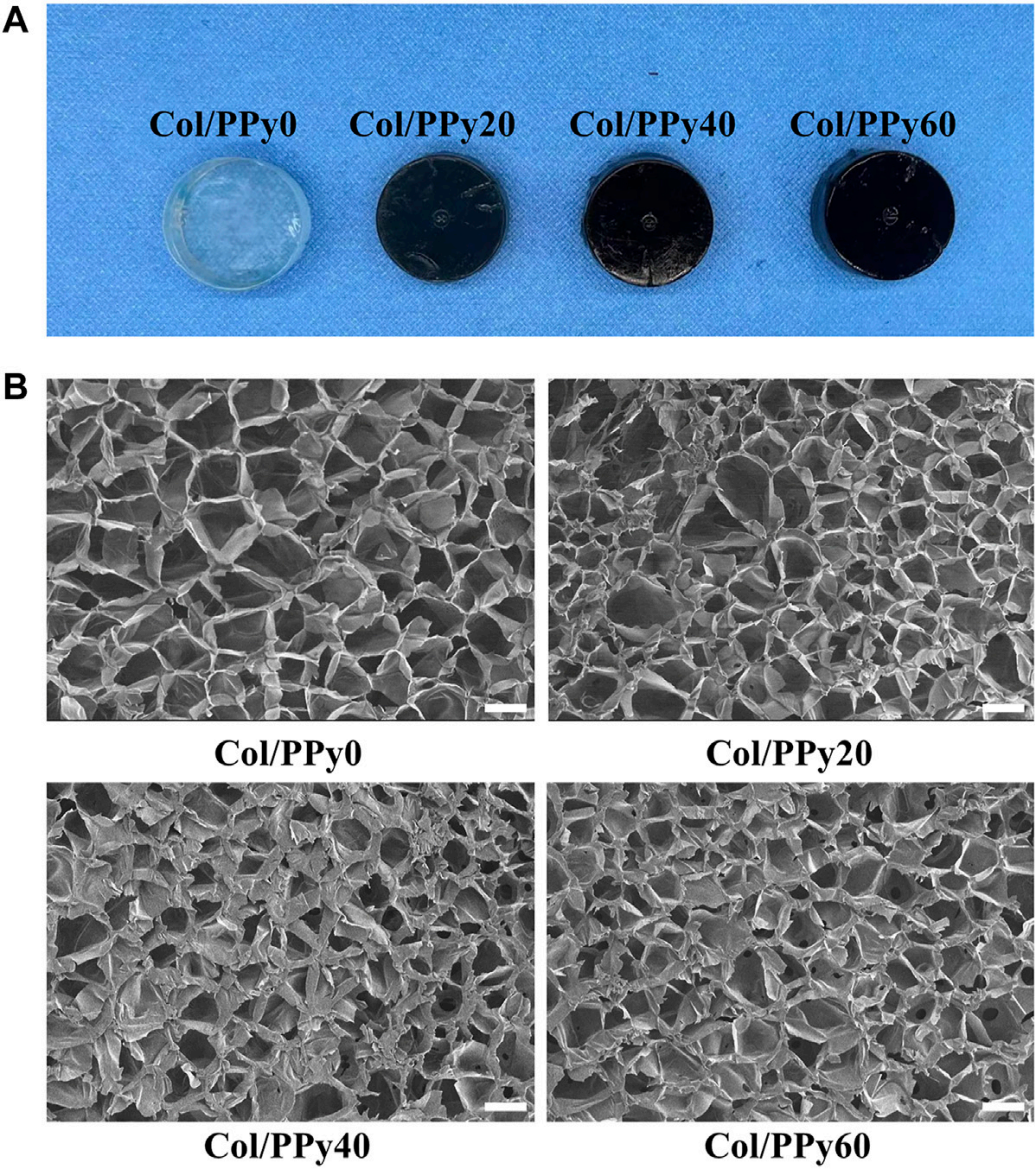
**(A)** Photographs of the various Col-PPy hydrogels; **(B)** SEM images of various Col-PPy hydrogels, scale bar 100 mm.

In this study, the pyrrole monomer and collagen were mixed uniformly, and then an oxidizing agent was introduced to the mixture to initiate the pyrrole polymerization reaction. The formed polypyrrole chain interpenetrated and intertwined with the collagen triple helix structure, resulting in a stable hydrogel network (Supplementary Figure S1). The cross-linking agent, genipin, could improve the stability of the prepared Col/PPy hydrogel and prolong the degradation ratio of the Col/PPy hydrogel. The images of Col/PPy hydrogels prepared at different pyrrole concentrations (i.e., 0 mM, 20 mM, 40 mM, and 60 mM) are shown in Figure 1A. As the concentration of pyrrole monomer increases, the color of the prepared hydrogel becomes darker. The increased content of PPy in the Col/PPy hydrogel improves the stiffness and conductivity of the hydrogel. The microscopic morphologies of Col/PPy hydrogels were observed using the SEM. Figure 1B shows the microporous structure inside the hydrogel with pore sizes ranging from 50 to 200 μm. As the content of PPy in the hydrogel increases, the microporous network structure of the hydrogel becomes more compact (Supplementary Figure S2). This could be because the more the PPy chains, the higher the degree of entanglement with collagen, which leads to a tighter hydrogel network structure (Luo et al., 2021). The relatively large pore size of the prepared hydrogels facilitates nutrient permeation, oxygen and carbon dioxide exchange, and promotes the growth, proliferation, expansion, and differentiation of cells (Yang et al., 2016; Zarei et al., 2021).

**FIGURE 2:**
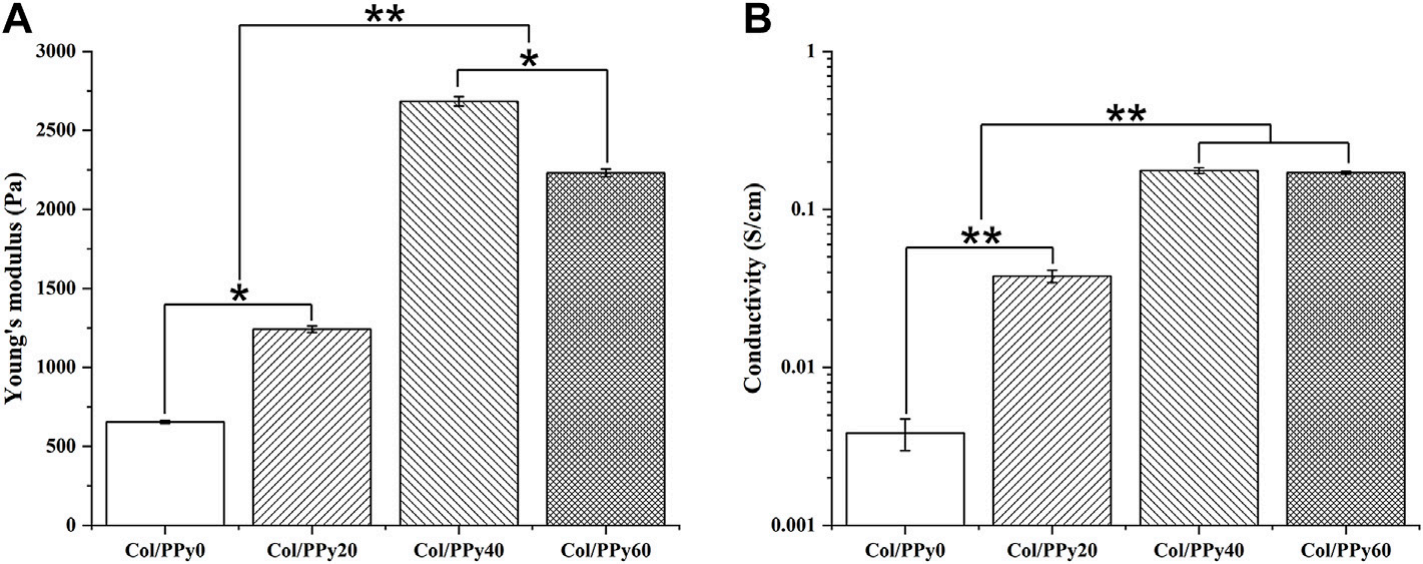
**(A)** Young’s modulus and **(B)** conductivity of various Col/PPy hydrogels.

The mechanical test results showed that the Young’s modulus of the prepared hydrogels was remarkably enhanced with the PPy content in the range of 0–40 mM. The Young’s moduli of Col/PPy0, Col/PPy20, and Col/PPy40 hydrogels were 654.5 ± 10.32 Pa, 1241.3 ± 20.4 Pa, and 2,684.5 ± 30.2 Pa, respectively (Figure 2A). However, the Young’s modulus of Col/PPy60 was approximately 20.3% lower than that of Col/PPy40. This is because the high PPy concentration and the oxidizing agent cause the mixed solution to react too fast (Min et al., 2018). The resulting heterogeneous PPy polymeric network reduces the structural stability of the hydrogel network. A study has suggested that the Young’s modulus value of hydrogel for nerve repair is in the range of spinal cord tissue modulus (100–3,000 Pa) (Jiang et al., 2017). Therefore, the prepared Col/PPy hydrogels (with PPy in the range of 0–60 mM) could provide good mechanical support for the repair of nerve cells and tissues.

The prepared hydrogel can simulate the conduction behavior of nerve cells and tissues (10^−2^–10^−1^ S/m) (Gabriel et al., 2009), which is beneficial in mediating electrical signals between tissues and accelerating the rate of nerve repair. The electrical conductivity of Col/PPy hydrogel was evaluated by electrical conductivity. The results (Figure 2B) show that an increase in PPy content enhances the electrical conductivity of the prepared Col/PPy hydrogels. The conductivity of Col/PPy40 hydrogel is the highest (0.176 ± 0.07 S/cm), which is 15 times that of Col/PPy0 (0.0038 ± 0.0009 S/cm). The conductivity of Col/PPy60 (0.1706 ± 0.0037 S/cm) and Col/PPy40 are almost the same. The results indicate that the conductivity of the Col/PPy hydrogel weakens when PPy exceeds 40 mM. This might be due to the instability and heterogeneity of the Col/PPy60 hydrogel network structure (Yang et al., 2016).

### *In Vitro* Degradation and Swelling Properties of Self-Assembling Cross-Linking Polymer Network Hydrogels

**FIGURE 3:**
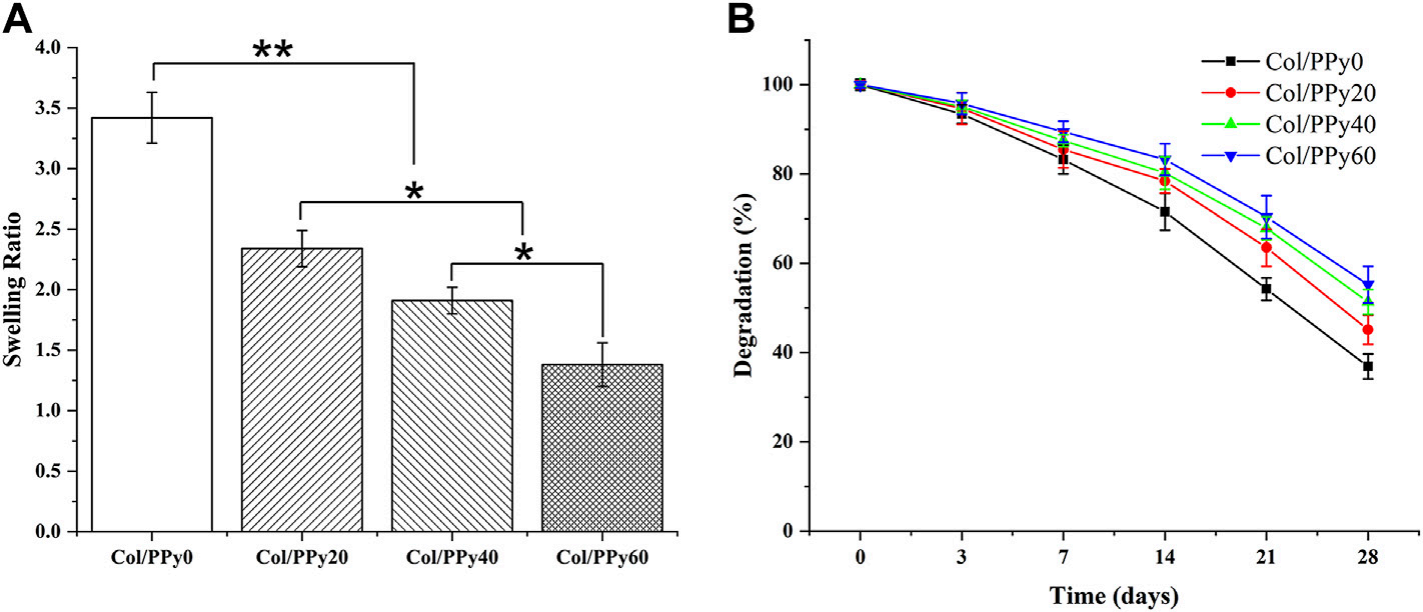
Swelling properties **(A)** and degradation curves **(B)** of Col/PPy hydrogels.

The hydrogel’s hydrophilic and hydrophobic components, as well as the cross-linking density, which has a significant impact on the adhesion and growth of cells on the hydrogel surface. Figure 3A shows the results of the swelling behavior of the prepared Col/PPy hydrogels. It suggests that the introduction of PPy into the collagen hydrogel reduces the swelling rate of the hydrogel. The swelling ratio of Col/PPy0 was approximately 2.48 times that of Col/PPy60. This might be because the hydrophobic PPy occupied the 3D space of the hydrogel, and the tight network caused by the secondary cross-linking of genipi reduced the water-binding capacity of the gel. The internal framework of Col/PPy hydrogel could not absorb water molecules after swelling to a certain degree. The swelling performance study suggests that the prepared hydrogel can provide a stable scaffold to support the growth of nerve cells in a humoral environment.

The biodegradation rate of hydrogel is an important indicator of its repair performance. The fast degradation rate of hydrogels causes the formation of voids in the damaged site, thus inhibiting repair, whereas the slow degradation of hydrogels hinders the formation of voids. Therefore, the degradation rate of the scaffold should match the healing rate of the injury site. The degradation results of the Col/PPy hydrogel are shown in Figure 3B. The prepared Col/PPy0 hydrogel sample had the fastest degradation rate, and the remaining weight was 36 ± 2.78% on the 28^th^ day. The degradation rate of the prepared collagen hydrogel was slower than that of self-assembly collagen hydrogels. After genipin cross-linking, the prepared hydrogel network density increased, and the anti-degradation ability improved. When the PPy content was increased, the degradation rate of the hydrogel decreased. On the 28^th^ day, the degradation residual weights of Col/PPy20, Col/PPy40, and Col/PPy60 were 45.11 ± 3.25%, 51.33 ± 2.78, and 55.24 ± 4.11%, respectively. This result indicates that the addition of PPy prolongs the degradation of the hydrogel. The PPy chains interpenetrated and entangled with the collagen, thus tightening the network structure of the hydrogel and improving its stability.

### Identification of Neural Stem Cells

**FIGURE 4:**
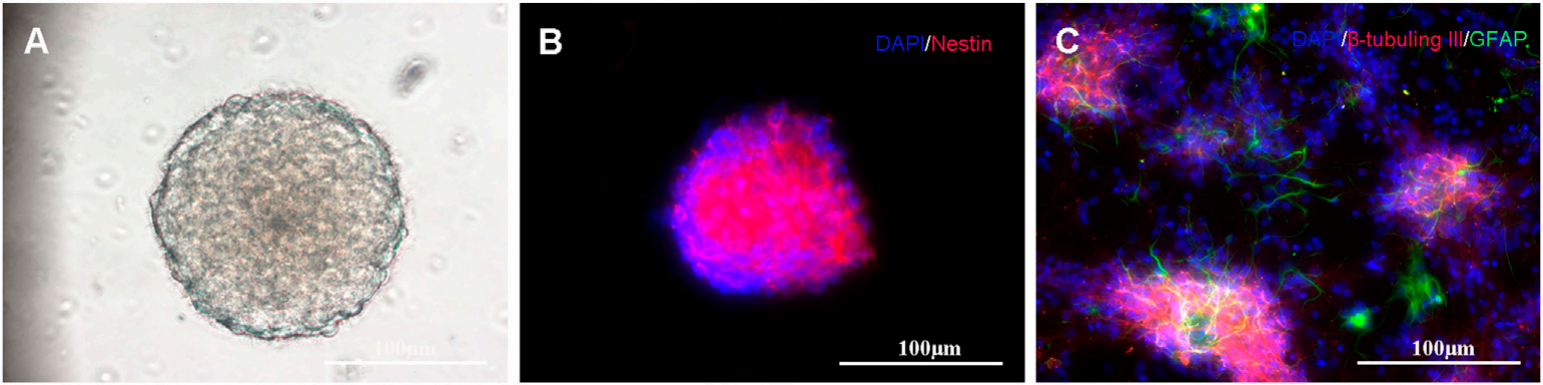
Primary culture and characterization of NSCs: **(A)** NSCs cultured in serum-free medium for 7 days; **(B)** Immunostaining of NSCs with nestin antibody (shown in red) and DAPI (shown in blue); and **(C)** When transferred to the differentiating state, the neurospheres expressed neuronal (β-tubulin III, shown in red) and astrocytic markers (GFAP, shown in green).

Self-renewal and multipotency are important characteristics of NSCs. Most isolated cells grow primarily as neurospheres and their formations are considered the gold standard for NSC identification (Azari et al., 2011; Louis et al., 2008), as shown in Figure 4A. The immunocytochemistry results indicate that the neurospheres are positively immunoreactive to nestin (Figure 4B). After neurospheres were induced to differentiate, they were stained positive for the neuronal marker β-tubulin III and astrocytic marker glial fibrillary acidic protein (Figure 4C).

### Cell Proliferation Experiments Under Different ES Conditions

**FIGURE 5:**
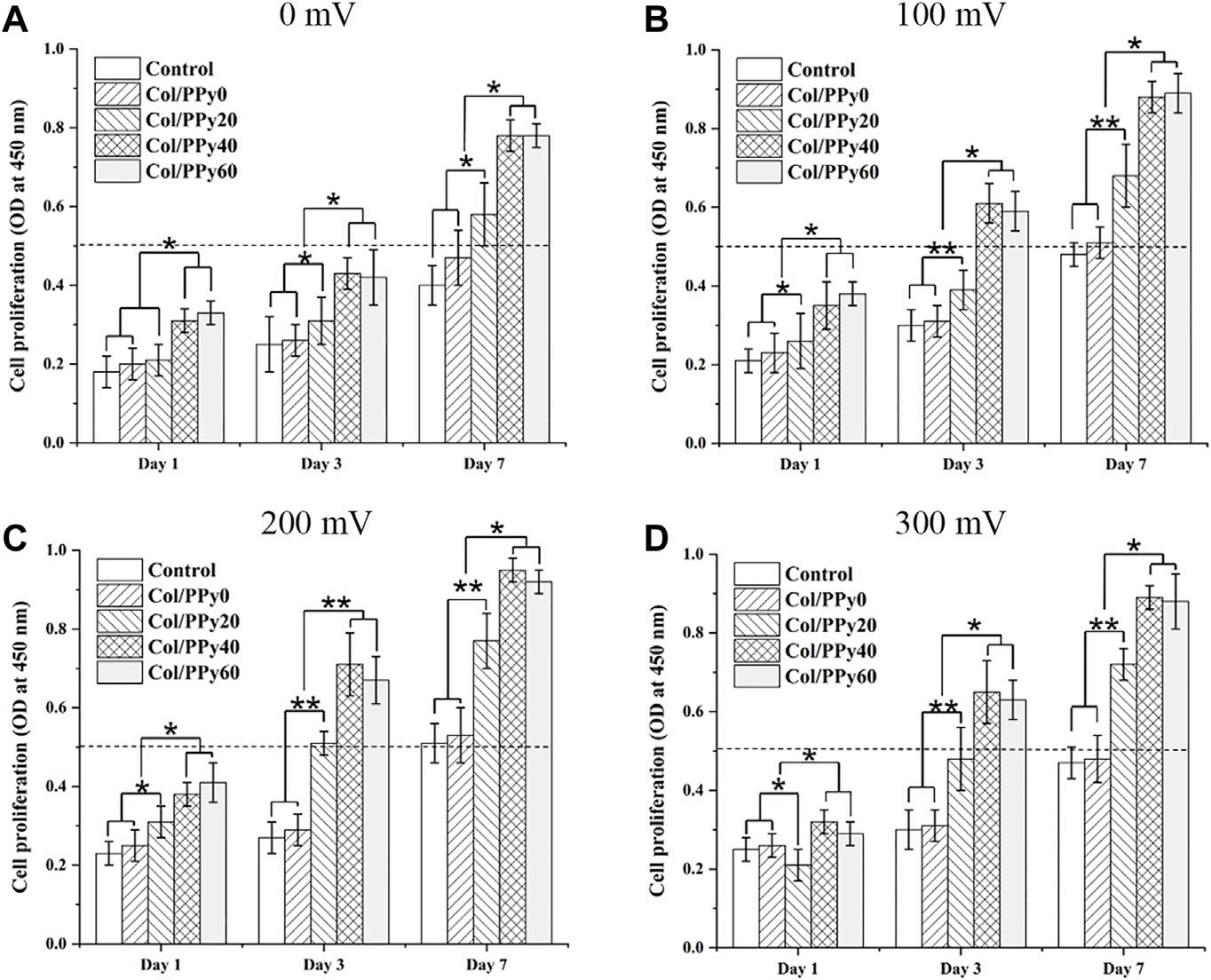
Cell proliferation assay. Proliferation of NSCs on different Col/PPy hydrogels on the first, third, and seventh days after electrical stimulation with different voltages **(A)**: 0 mV, **(B)** 100 mV, **(C)** 200 mV, **(D)** 300 mV).

To study the effect of ES of different voltages on the proliferation of NSCs, the proliferation of NSCs on Col/PPy0, Col/PPy20, Col/PPy40, and Col/PPy60 was detected using the CCK-8 assay. The results show that without the ES treatment (Figure 5A), all prepared Col/PPy hydrogels had good biocompatibility with NSCs after 7 days of co-incubation. When the ES treatment was applied at 100 mV (Figure 5B) on day 7, the Col/PPy40 and Col/PPy60 sample groups exhibited the highest OD value, followed by the Col/PPy20, Col/PPy0, and control groups. When the ES treatment was applied at 200 mV (Figure 5C) and 300 mV (Figure 5D) on day 7, all sample groups exhibited a trend similar to that of ES at 100 mV (Figure 4B). These results suggest that both the Col/PPy40 and Col/PPy60 hydrogels can effectively promote NSCs proliferation when compared to other groups, regardless of the presence or absence of the ES treatment. When the ES treatment was applied at 200 mV on day 7, the Col/PPy40 sample group exhibited the highest OD value compared to the other groups or ES conditions. Therefore, the Col/PPy40 hydrogel was selected as the most suitable candidate carrier for NSCs differentiation and proliferation, and the ES was set to 200 mV.

### Collagen/Polypyrrole40 Coupled ES to Promote Neural Stem Cells Viability

**FIGURE 6:**
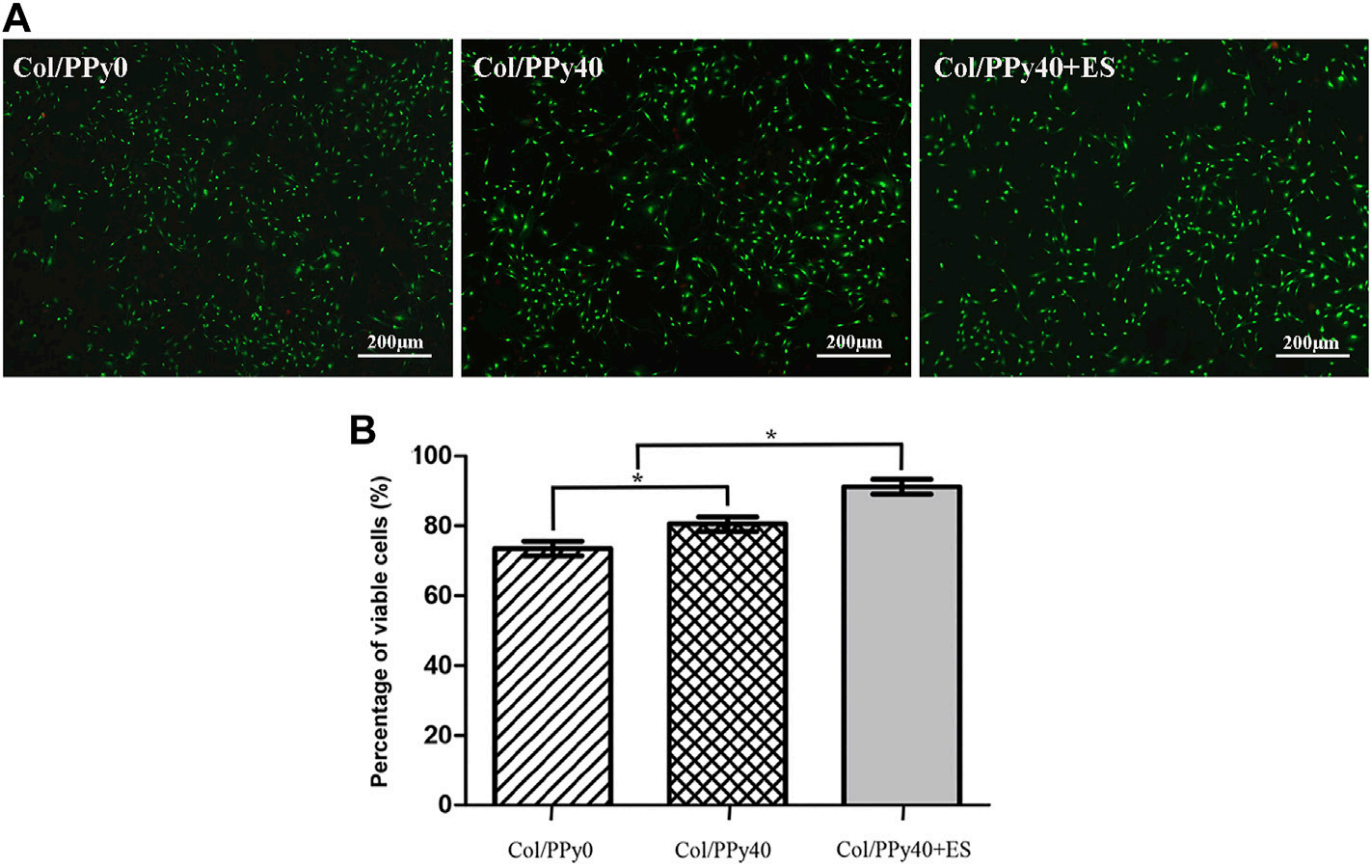
**(A)** Live/dead fluorescence staining images of cells differentiated from NSCs in Col/PPy0, Col/PPy40, and Col/PPy40 + ES (200 mV) groups on day 3; live cells are shown in green fluorescence and dead cells are shown in red fluorescence; **(B)** Percentage of viable cells cultured in different groups on day 3.

To test the cytotoxicity of SCPN and the effect of ES on NSCs, the viability of NSCs cultured in the prepared hydrogel groups was assessed on day 3 using a live/dead assay. The live cells were stained green, and the dead cells were stained red, as indicated by the confocal images (Figure 6A). The differentiated cells from NSCs incubated with Col/PPy40 + ES had the highest green fluorescence intensity among all sample groups. The highest percentage of viable NSCs was the Col/PPy40 + ES group (91.2 ± 4.8%), followed by Col/PPy40 (80.5 ± 4.7%), and Col/PPy0 (73.5 ± 4.8%) groups, as shown in Figure 6B.

These results indicate that conductive polymer PPy with ES treatment can promote the viability of NSCs. PPy is the most investigated conductive polymer in SCI owing to its high conductivity and ease of incorporation of biomolecule (Raynald et al., 2019). In the prepared collagen base hydrogels, PPy increased the stability of hydrogel scaffolds and relatively easy-adhere hydrogel surfaces, which was beneficial for cell growth (Ketabat et al., 2017). PPy also increased the conductivity of the hydrogel when ES treatment was applied, thus promoting the conduction of electrical signals between cells, which is critical for NSCs self-renewal and survival (Supplementary Figure S3).

### Collagen/Polypyrrole40 Coupled ES to Promote Neural Stem Cells Differentiation

**FIGURE 7:**
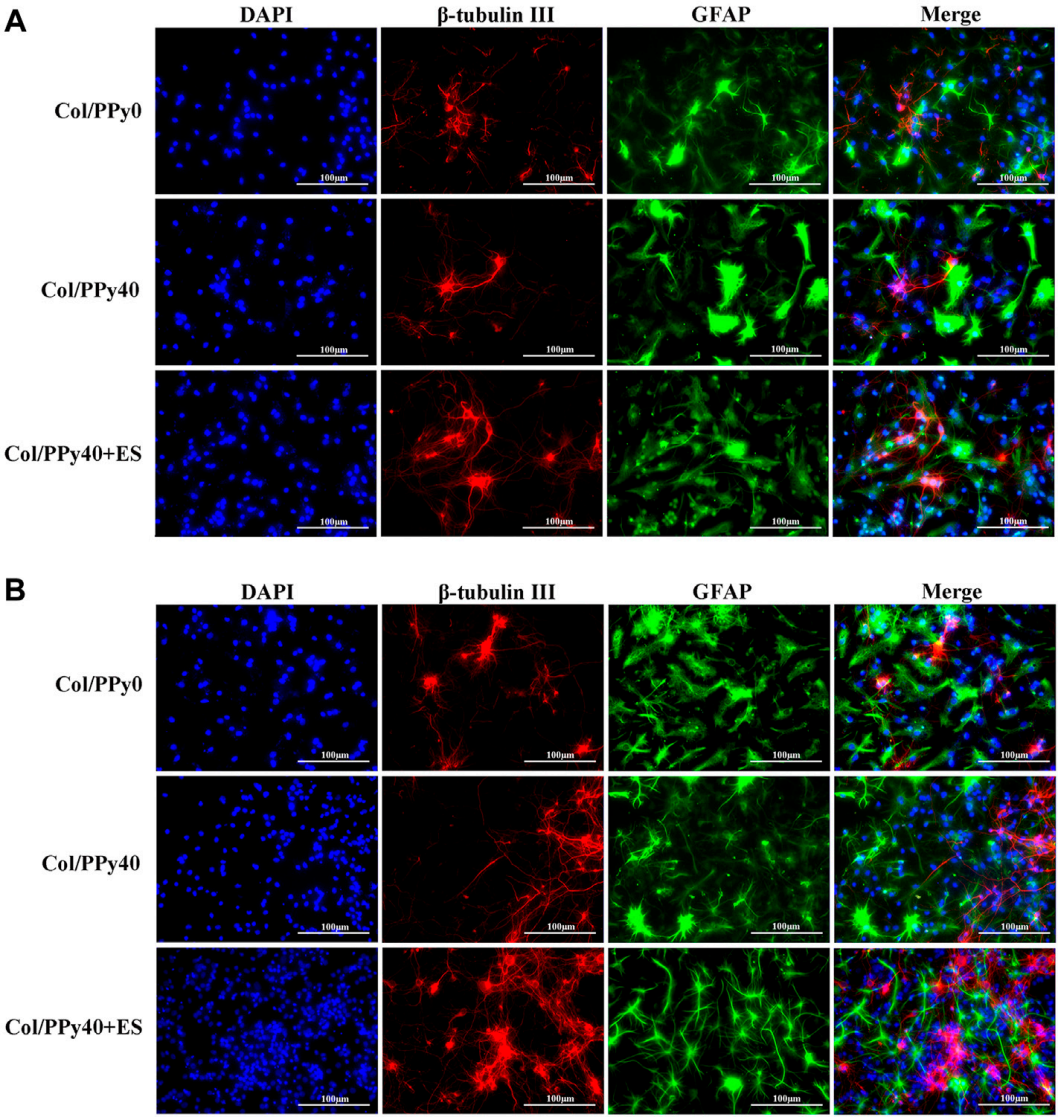
Confocal microscopy images of NSCs differentiation with the prepared hydrogels into β-tubulin III positive cells (for neurons, shown in red) and GFAP positive cells (for astrocytes, shown in green) on day 3 **(A)** and day 7 **(B)**. All cell nuclei were DAPI stained (shown in blue).

**FIGURE 8:**
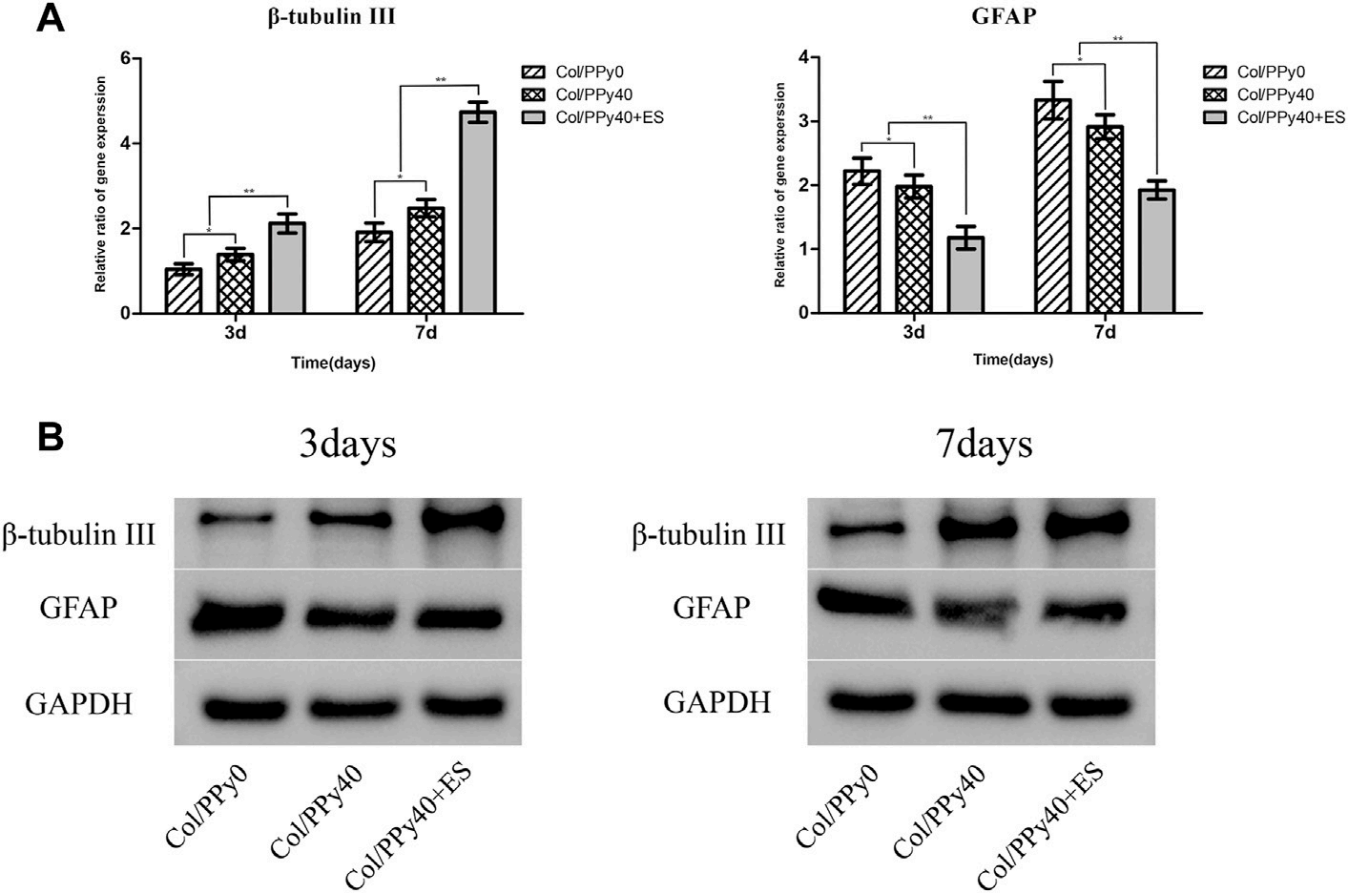
Gene **(A)** and protein **(B)** expression levels of β-tubulinIII and GFAP of the NSCs differentiated in Col/PPy0, Col/PPy40 and Col/PPy40 + ES (200 mV) groups on day 3 and day 7.

NSCs have the potential to differentiate into neurons and astrocytes, and the differentiation of NSCs is affected by intracellular and extracellular factors. To investigate the effect of conductive hydrogel coupled with ES on NSCs differentiation, we qualitatively and quantitatively analyzed different groups of hydrogel-cocultured NSCs after 3 d and 7 days using immunocytochemical analysis, qPCR, and Western bolt. The acquisition of neuronal- and astrocyte-type cells was evaluated using the expression of the neuron-specific marker (β-tubulin III) and astrocyte-specific marker (GFAP), respectively. Compared to cells grown on Col/PPy0, cells cultured on Col/PPy40 scaffolds, with or without ES, exhibited significantly higher β-tubulin III expression and lower GFAP expression. The highest expression of β-tubulin III was observed on Col/PPy40 scaffolds in the presence of ES (Figure 7). qPCR was performed to quantitatively evaluate the differentiation behaviors of NSCs. The gene expression data suggest that NSCs in Col/PPy40 + ES had significantly higher percentages of neurons and lower percentages of glial cells than those in the other groups after 3 d and 7 days (Figure 8A), which is consistent with the immunostaining results. NSC differentiation was also observed in the Western blot analysis of β-tubulin III and GFAP protein levels, and the results were consistent with those of qPCR (Figure 8B). These results indicate that the combined application of Col/PPy40 and ES maximally induces the differentiation of NSCs into neurons and inhibits the differentiation of NSCs into astrocytes. ES plays an important role in inducing appropriate stem cell responses and has great potential to modulate stem cell proliferation, self-renewal, and differentiation. Recent studies have confirmed that the differentiation of cultured NSCs into neurons is directly related to the regulation of ES (Cheng et al., 2021). ES may be directly involved in the up-regulated expression of L-type voltage-gated calcium channels (L-VGCCs), which are essential for the activation of Ca^2+^-dependent pathways through Ca^2+^ influx (Wu et al., 2019). It has been reported that Ca^2+^-dependent phosphatidylinositol 3 kinase (PI3K)-protein kinase B (Akt) and mitogen-activated protein kinase (MAPK) signaling pathways are involved in the ES-induced neurogenesis of NSCs (Wu et al., 2021). Therefore, a cellular microenvironment composed of a conductive matrix is beneficial to the transmission of electrical signals to cells (Hu et al., 2014; Ferrigno et al., 2020). Our results indicate that Col/PPy40 can serve as an efficient interface for electrical transmission, and when coupled with ES, it can provide an ideal growth microenvironment for NSCs, regulate the differentiation of NSCs, and promote the main differentiation of NSCs into neurons.

### Collagen/Polypyrrole40 Coupled ES to Promote Neurite Outgrowth of Neural Stem Cells-Derived Neurons

**FIGURE 9:**
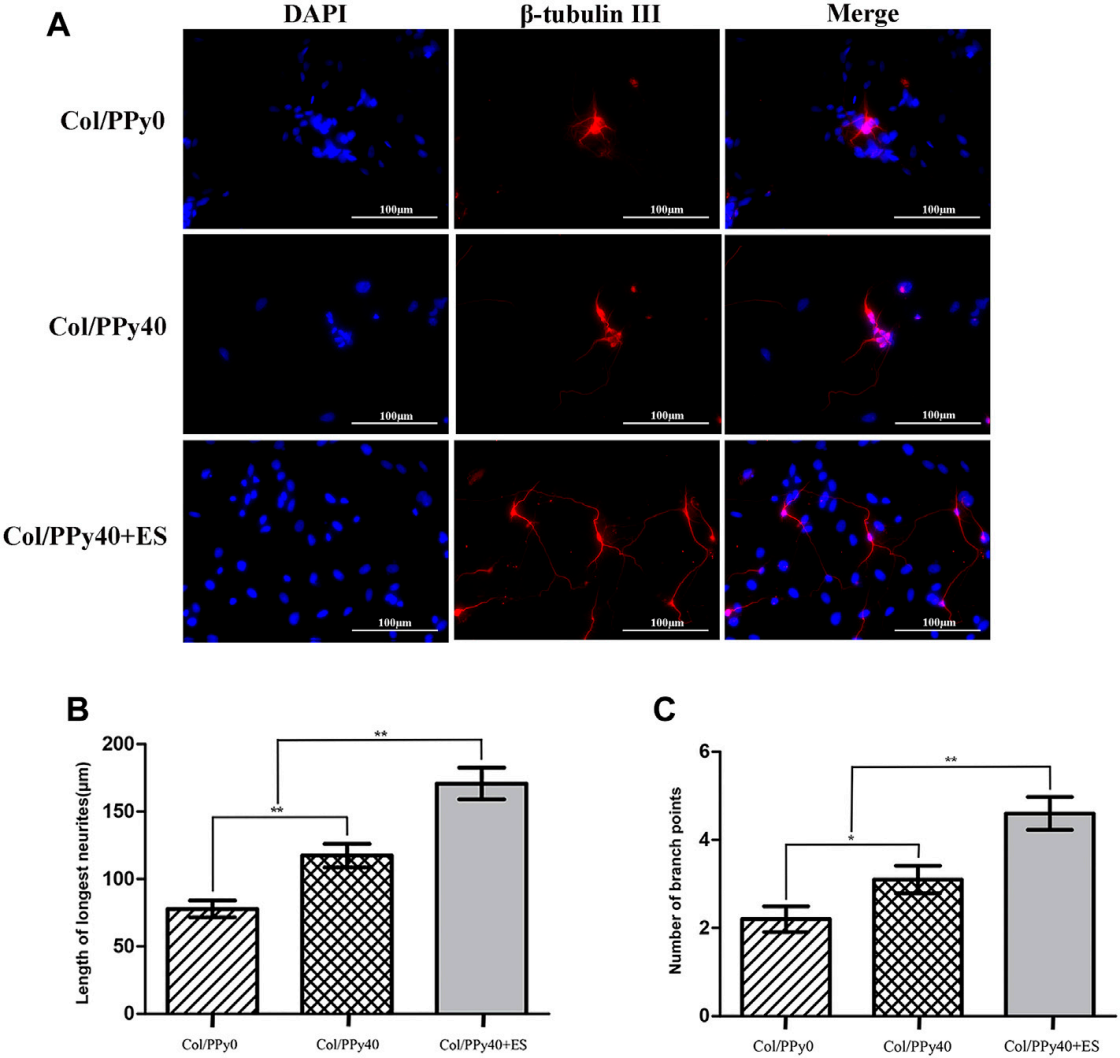
Outgrowth of NSC-derived neurons in Col/PPy0, Col/Ppy40, and Col/PPy40 + ES (200 mV) groups. **(A)** Representative fluorescent images of NSC-derived neurons cultured β-tubulin III was stained red, and the nuclei were stained blue; **(B)** Length of the neurites in different groups. **(C)** Number of branch points in different groups.

To investigate the effect of Col/PPy40 combined with ES treatment on neurite outgrowth after NSCs differentiation, immunocytochemical analysis was used to quantitatively analyze the length of neurites and the number of branch points of NSC-derived neurons. As shown in Figure 9A, the morphological characteristics of neurons differentiated from the three groups of hydrogels were different. The longest neurite length (170.8 ± 37.2 μm) was observed in the Col/PPy40 + ES hydrogel group, followed by the Col/PPy40 group (117.4 ± 27.6 μm), and the shortest neurite length (77.8 ± 19.7 μm) was observed in the Col/PPy0 group, as shown in Figure 9B. The numbers of branch points in the Col/PPy40 + ES group (4.7 ± 1.2) were significantly higher than those in the Col/PPy40 (3.1 ± 1.0) and Col/PPy0 (2.2 ± 0.9) groups, as shown in Figure 9C, which is consistent with the neurite length results.

In the nervous system, electrical signals can promote the interconnection between neurons, and affect the development and maturation of nerves. A recent study found that autophagy primarily through mTOR signaling is an important pathway in ES-promoted neurite outgrowth (He et al., 2021). Fibronectin plays a crucial role in cell attachment and neurite outgrowth. ES can alter the local electric fields of the extracellular matrix, thereby increasing the absorption of fibronectin into the conductive scaffolds (Molino et al., 2012). ES can also change the cell membrane potential and induce cell membrane depolarization, thus promoting neurite growth (Goganau et al., 2018). Our results also suggest that although Col/PPy40 promote neurite elongation, this elongation promotion was further enhanced when ES acted on the Col/PPy40 hydrogel. Thus, the interaction between the ES and Col/PPy40 hydrogel may have further increased the material surface’s electrical activity for promoting neurite elongation.

## Conclusion

In this study, we prepared a series of collagen-PPy-based hydrogels to load NSCs for SCI therapy. The characterization results indicate that Col/PPy40 hydrogel has the most optimized mechanical strength, electrical conductivity, degradation, and swelling properties. All the prepared hydrogels had little cytotoxic effects on the NSCs, and Col/PPy40 + ES exhibited the highest cell proliferation activity and viability compared with other hydrogel samples. The directional differentiation abilities of the prepared hydrogels were investigated using immunocytochemical analysis, qPCR, and Western bolt. The differentiation studies demonstrate that Col/PPy 40 + ES can effectively promote NSCs differentiation into neurons and inhibit the differentiation of NSCs into astrocytes. The results suggest that Col/PPy40, as a conducting microenvironment, combined with ES could have significant applications in neural tissue engineering. We envisage conducting *in vivo* investigations in future work.

## Data Availability

The original contributions presented in the study are included in the article/Supplementary Material, further inquiries can be directed to the corresponding authors.
