# Supplementary material for: Conductive Collagen-Based Hydrogel Combined With Electrical Stimulation to Promote Neural Stem Cell Proliferation and Differentiation
Source: Front Bioeng Biotechnol. 2022 Jun 17;10:912497. doi: 10.3389/fbioe.2022.912497 (PMC9247657; doi:10.3389/fbioe.2022.912497)
Supplement: Supplementary file 1 [file Table1.DOC]

**Supporting information**

**Conductive Collagen-Based Hydrogel Combined with Electrical Stimulation to Promote Neural Stem Cell Proliferation and Differentiation**

Xinzhong Xu^a1^, Lin Wang^b1^, Juehua Jing^a^, Junfeng Zhan^a^, Chungui Xu^a^, Wukun Xie^a^, Shuming Ye^a^, Yao Zhao^a^, Chi Zhang^c*^, Fei Huang^a*^

a Department of Orthopaedics, The Second Affiliated Hospital of Anhui Medical University, Hefei 230000, Anhui, China

b Department of Orthopaedics, Yijishan Hospital of Wannan Medical College, Wuhu 241000, Anhui Province, China

c Department of Orthopaedics, The Fourth Affiliated Hospital of Anhui Medical University, Hefei, Anhui, China

The authors declare no conflicts of interest.

1 These authors have equal contribution to this work and should be considered as co-first author.

* Corresponding author, Fei Huang, Department of Orthopaedics, The Second Affiliated Hospital of Anhui Medical University, No 678 Furong Road, Jingkai District, Hefei 230000, Anhui Province, People’ s Republic of China. Email: [huangfei3738@126.com](mailto:Yinzongsheng2018@126.com). Chi Zhang, Department of Orthopaedics, The Fourth Affiliated Hospital of Anhui Medical University, No100 Huaihai Road, Xinzhan District, Hefei 230000, Anhui Province, People’ s Republic of China. Email: [563010754@qq.com](mailto:Yinzongsheng2018@126.com)

Tel: + 86 551 63869501; fax: + 86 551 63869501

1. **The rheology properties of the hydrogel**

**
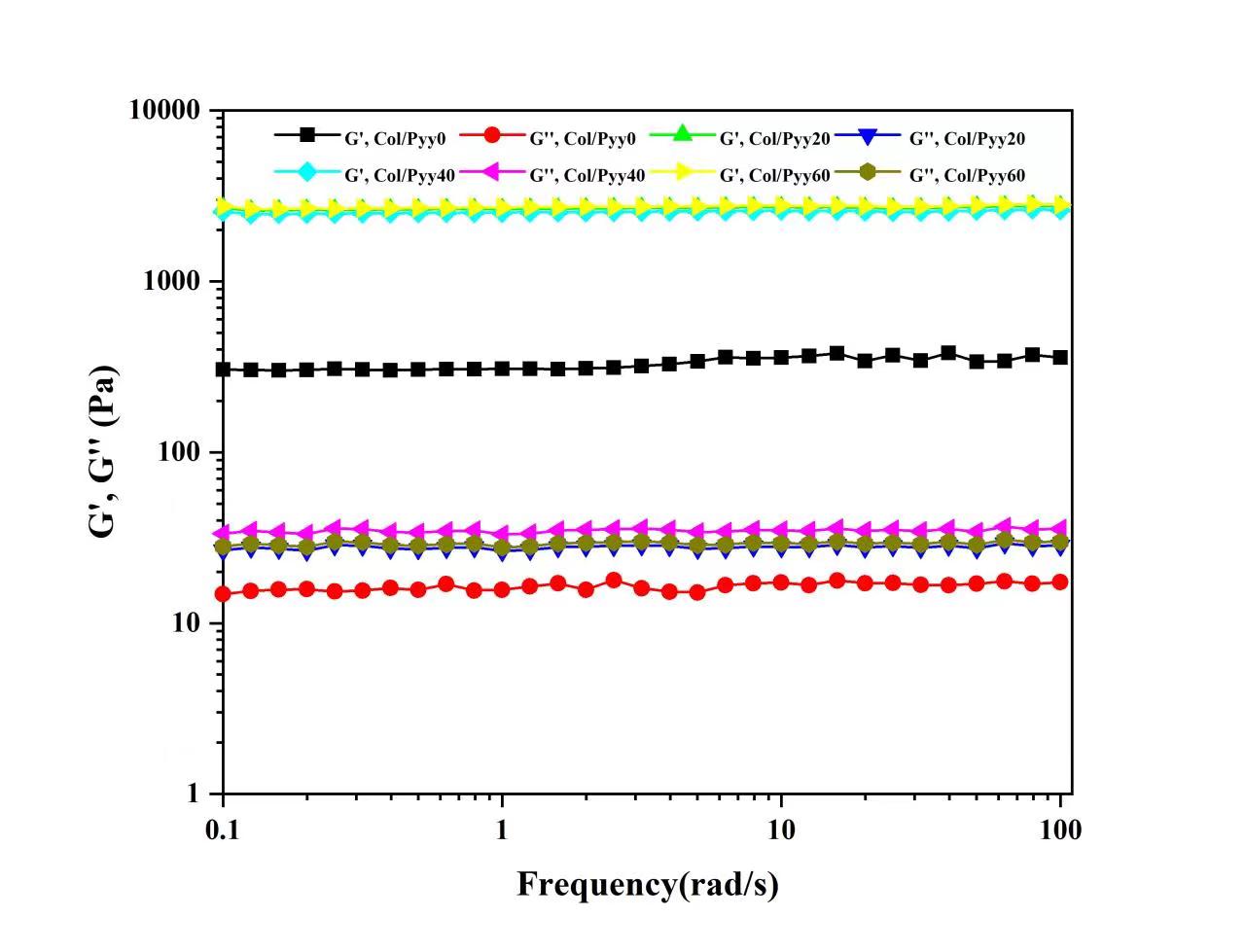
**

**Fig. S1**.The rheology properties of the hydrogel

1. **The porosity of the prepared hydrogels**

|  | Porosity（%, mean ± SD） |
| --- | --- |
| Col/Pyy0 | 84.72±2.26 |
| Col/Pyy20 | 81.83±1.58 |
| Col/Pyy40 | 79.49±0.83 |
| Col/Pyy60 | 76.14±2.05 |

**Fig. S2**. he porosity of the prepared hydrogels

1. **Neural Colony-forming Cell Assay**

**
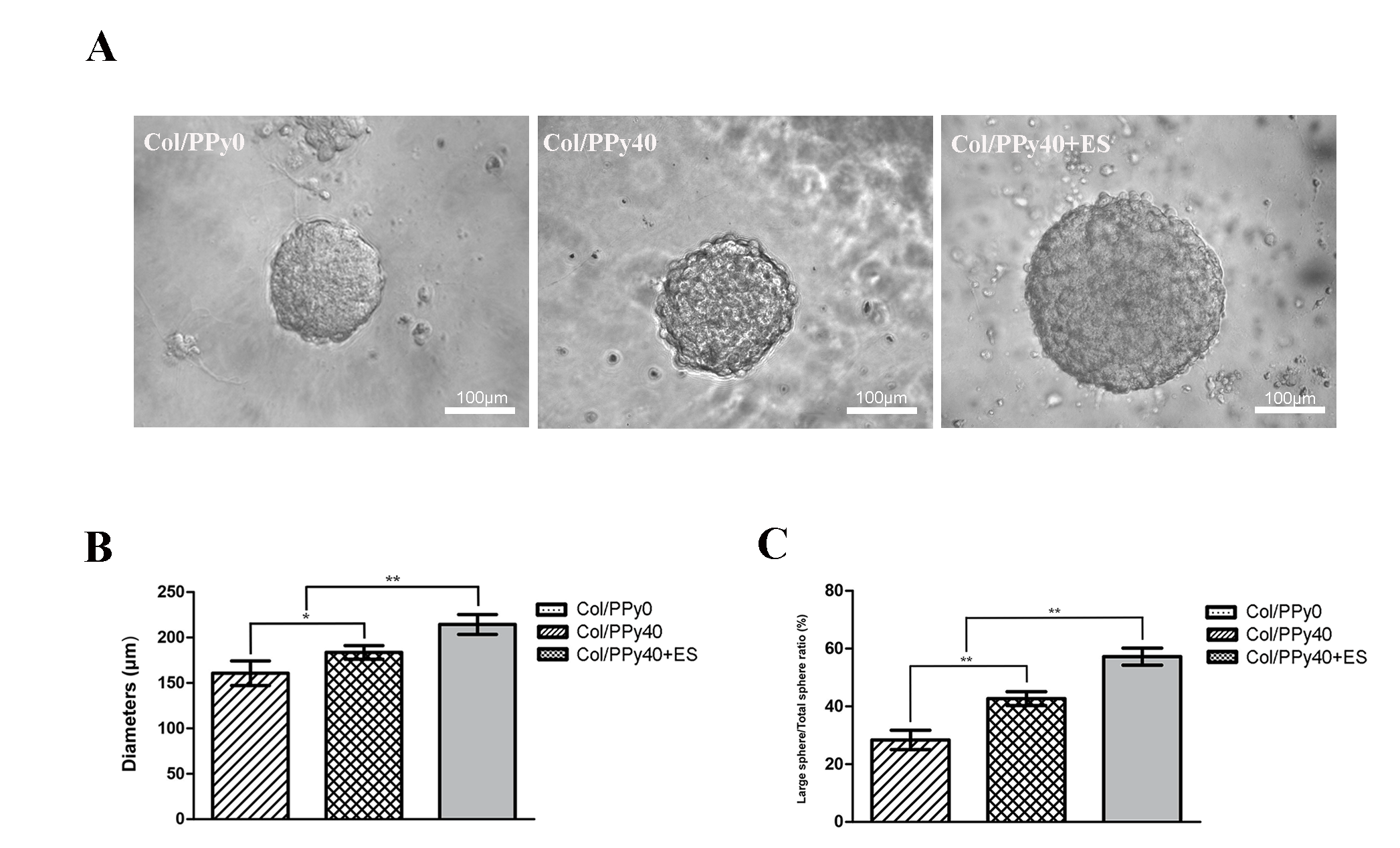
**

**Fig. S3**. Assessment of the colony-forming ability of NSCs cultured on different hydrogels after 7 days. (A) Pictures of neurospheres in the Col/PPy0, Col/PPy40 and Col/PPy40+ES groups; (B) Diameter of neurospheres in the Col/PPy0, Col/PPy40 and Col/PPy40+ES groups; (C) Ratio of large neurospheres in the Col/PPy0, Col/PPy40 and Col/PPy40+ES groups.
